# Application of Collodion in Cholera

**Published:** 1867-09

**Authors:** 


### *Application of Collodion in Cholera.*

Dr. Drouet, of La Grand-Montrouge, maintains that the external application of collodion will arrest the premonitory diarrhœa, and afford an excellent means of restoring warmth in confirmed cholera. He uses a mixture of collodion 6 parts, castor oil 1 part, smeared over the abdomen, and covered with cotton wool. The evaporation of the ether at first causes a sensation of cold, but in a few minutes this is followed by a feeling of warmth, which increases in intensity, without, however, becoming so intense as to cause distress. This application, he says, will certainly arrest the progress of the disease, if used during the first hours of the attack, and provided it be not of an extremely violent nature. —(*L'Union Médicale.*)—*N. Y. Medical Journal.*

